# A Late-Onset and Mild Phenotype of Mitochondrial Complex I Deficiency Due to a Novel Reported Variant Within the *ACAD9* Gene

**DOI:** 10.3390/ijms26157128

**Published:** 2025-07-24

**Authors:** Anna Gaelle Giguet-Valard, Samira Ait-El-Mkadem Saadi, Sophie Duclos, Didier Lacombe, Rémi Bellance, Nadège Bellance

**Affiliations:** 1 Department of Rare Neurological and Neuromuscular Diseases—Martinique University Hospital, 97261 Fort-de-France, Martinique, France; anna.giguet-valard@chu-martinique.fr (A.G.G.-V.);; 2 BIOSPHERES—AREBio Laboratory, 97233 Schoelcher, Martinique, France; 3 Genetics Laboratory Department Mitochondrial Pathologies, University Hospital Center of Nice, 06000 Nice, France; 4 INSERM U1211, Rare Diseases: Genetics and Metabolism, 33076 Bordeaux, France; 5 Biological and Medical Sciences Research Department, University of Bordeaux, 33076 Bordeaux, France

**Keywords:** mitochondrial cytopathology, variant of uncertain significance, complex I, respiratory chain

## Abstract

Acyl-CoA dehydrogenase 9 deficiency is considered as a rare neuromuscular syndrome with an autosomal recessive transmission. The ACAD9 protein presents two essential functions, i.e., the limiting step enzyme of the fatty acid β-oxidation pathway and one of the complex’s compounds involved in the respiratory chain complex I assembly. Thus, loss-of-function mutations are known to convey mitochondrial cytopathologies. A patient with a mild and late-onset phenotype, suffering from exercise intolerance and hypertrophic cardiomyopathy, was diagnosed as a compound heterozygote of the *ACAD9* gene. The first c.1240C> T p.Arg414Cys variant has been previously reported and is known to be responsible for *ACAD9* deficiency. However, the second c.1636G> A p.Val546Met variant has never been described. The goal was to investigate the eventual pathogenicity of this new genetic variant. For this purpose, molecular cloning was generated to express the *ACAD9* gene with the V546M variant in a cell line (ACAD9mut) and compared to cells expressing the wild-type *ACAD9*. Then, the mitochondrial respiration, ATP production, the mitochondrial network, and the oxidative phosphorylation’s composition were investigated to reveal the effects of the V546M variant. While avoiding to affect the amount of the respiratory chain’s complexes, the new *ACAD9* variant was entirely responsible for reducing over 50% of the mitochondrial complex I activity.

## 1. Introduction

Acyl-CoA dehydrogenase 9 (ACAD9) deficiency is a Mendelian disease linked to alterations in the *ACAD9* gene functions, transmitted in an autosomal recessive pattern (OMIM # 611126) [1]. This disease has a wide variety of clinical expressions whose characteristics mainly involve the cardiovascular and neuromuscular systems [2]. Sometimes, hepatic, metabolic, or hematological disorders may be associated.

The typical clinical phenotype describes the infantile onset of acute metabolic acidosis, hypertrophic cardiomyopathy, and muscle weakness associated with a deficiency of mitochondrial complex I activity in the muscles, liver, and fibroblasts [3,4]. Severely affected individuals may have encephalomyopathy. Reye-like syndrome episodes can occur [5]. Individuals who survive past early childhood often have intellectual disability and may develop seizures. Mildly affected individuals usually experience exercise intolerance (nausea and extreme fatigue in response to physical activity). They have low muscle tone (hypotonia) and skeletal muscles weakness [6,7]. Actually, these patients are characterized by thrombocytopenia, hypoglycemia, elevated plasma ammonia; liver transaminases, serum lactate, or lactate dehydrogenase, and prothrombin time increased. Some patients can have hypoketotic dicarboxylic aciduria or elevated long-chain acyl-carnitine species [7].

ACADs are a family of mitochondrial enzymes catalyzing the initial rate-limiting step in the β-oxidation of fatty acids (FAOs). Among the eight human ACAD proteins already described, four are involved in the β-oxidation of straight-chain fatty acids including short-chain (SCAD), medium-chain (MCAD), long-chain (LCAD), and very-long-chain acyl-CoA dehydrogenase (VLCAD), and four are involved in the degradation of branched-chain fatty acids [8]. The straight-chain specific ACADs have different but overlapping substrates. The branched-chain specific ACADs catalyze substrates with different configurations of the hydrocarbon chain. The dehydrogenase activity of ACAD9 is specific to palmitoyl-coenzyme A (C16:0) and stearoyl-coenzyme A (C18:0) [8,9,10].

Studies on c. elegans revealed that a very-long-chain specific ACAD, ACDH-12, was an assembly factor of the respiratory complex I. ACDH-12 knockdown in nematodes did not exhibit any change in body fat content, suggesting that ACDH-12 may not be crucial to fatty acid oxidation. Interestingly, sequence analysis showed a high homology between ACDH-12 and the human *ACAD9* [11]. Several knockout mouse models were developed [12]. Homozygous knockout appeared to be lethal. Cardiac-specific ACAD9-deficient animals had severe neonatal cardiomyopathy and died at 17 days of age, with severe complex I mitochondrial deficiency. Muscle-specific mutants were viable but exhibited muscle weakness [12].

The *ACAD9* gene was identified by the large-scale random sequencing of human dendritic cell cDNA library. It has around 47% amino acid (AA) identity and 65% similarity with human very-long-chain acyl-CoA dehydrogenase [13].

The *ACAD9* gene is localized at 3q21.3 and has 18 exons. Alternate splicing are reported to induce at least five transcript variants. The major transcript (NM_014049.5) encodes for the longest protein (NP_054768.2) that contains 621 amino acids with a molecular mass of 68 kDa. The ACAD9 protein presents a transit peptide domain extended from the first AA to the thirty-seventh AA. It is a homodimer whose activity with a broad range of substrates and a greater specificity for long-chain unsaturated acyl-CoA [10]. The active site seems to be localized at glutamic acid E426 [9]. It is a proton acceptor site whose co-factor is flavin adenine dinucleotide (FAD) [2,4]. The ACAD9 protein is mainly organized in an N-terminal dehydrogenase domain (from AA38 to AA453) and a C-terminal vestigial domain (from AA487 to AA587). The protein is also a mitochondrial complex I assembly factor, which plays a role in oxidative phosphorylation. Thus, the ACAD9 protein interacts with NDUFAF1 (NADH: Ubiquinone Oxidoreductase Complex Assembly Factor 1) and ECSIT (Evolutionarily Conserved Signaling Intermediate In Toll Pathway) as part of the mitochondrial complex I assembly (MCIA complex). It also interacts with transmembrane protein family members (TMEM126B, TMEM186, and TIMMDC1) and an oxidase COA1 [14,15,16,17].

Fatty acid β-oxidation (FAO) and oxidative phosphorylation (OXPHOS) are mitochondrial metabolic processes responsible for energy production. FAO is an iterative process according to which four different enzymes successively execute the oxidation first (also known as the rate-limiting step), hydration, oxidation again, and thiolysis of their substrates. The final product of β-oxidation is acetylCoA than can enter the Krebs cycle.

In OXPHOS, energy production requires substrates, also known as reduced equivalents (NADH and FADH_2_), and a coupling between the respiratory chain constituted of four complexes (I, II, III and IV) and the F_1_F_0_-ATP synthesis (complex V). An alteration in the mitochondrial respiration affects the ATP production. FAO and the OXPHOS system are also linked by the reduced equivalents. β-oxidation recycles, i.e., reduces, NAD^+^ and FAD into NADH and FADH_2_ that are oxidized by the complexes I and II of the respiratory chain. When the biogenesis of the complex I occurs, ACAD9 switches from its role in FAO to an MCIA factor by interacting with ESCIT and being unable to fix FAD [18]. This mechanism coordinates ACAD9 involvement in the β-oxidation and the OXPHOS system.

ACAD9 deficiency is one of the so-called “riboflavin-sensitive” deficiencies [19]. Therefore, riboflavin, also known as vitamin B2, is currently the treatment of choice, because of its ability to increase the FAD content within the mitochondrial matrix. Haack et al. demonstrated that riboflavin treatment administered to a congenital case resulted in normal development at least up to 5 years of age [20]. A French National Protocol for the Diagnosis and Care of Medium Chain Fatty Acid Acyl-CoA Dehydrogenase Deficiencies, with other mitochondrial fatty acid β-oxidation alterations, including ACAD9 deficiencies, was validated in June 2021. It reports expert recommendations, as the rarity and phenotypic variability of the disease explain the difficulties in obtaining the scientific demonstration of the treatments’ effectiveness.

We have identified a patient affected by a myopathy sensitive to riboflavin with the presence of two variants of the *ACAD9* gene. This patient harbors a mild and late-onset phenotype, and her genotype reveals a compound heterozygote status associated with a c.1240C> T p.Arg414Cys class 5 variant, described by Repp et al. [7] and a c.1636G> A p.Val546Met class 3 or 4 variant, which has never been reported nor described. However, the change of valine to leucine at position 546 creates conflicting classifications of pathogenicity [21].

In our study, we performed functional experiments to bring evidence on the pathophysiological impact of this new genetic variant regarding the efficiency of the OXPHOS system.

## 2. Results

### 2.1. Compound Heterozygosity Is Associated with Mitochondrial Disturbance

**Figure 1:**
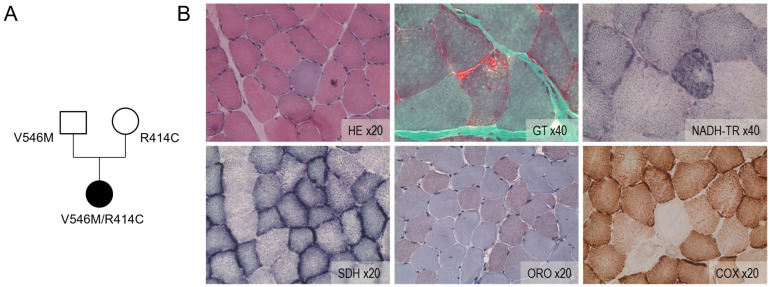
The histology of the patient’s muscle biopsy. (**A**) The family tree of the ACAD9 deficient patient (represented in dark symbol) shows the maternal and paternal genetic variants transmitted. (**B**) The histological sections obtained with hematoxylin and eosin stain (HE ×20) or Gomori trichrome (GT ×40) show few ragged red fibers. The activities of the mitochondrial OXPHOS complexes I and II were assayed for the patient sample via nicotinamide adenine dinucleotide dehydrogenase–tetrazolium reductase (NADH-TR ×40) and succinate dehydrogenase (SDH ×20). Muscle sections were stained with oil red O to visualize fat (ORO ×20), revealing an excess of lipid storage in fibers. The activity of the cytochrome oxidase (complex IV) was measured for muscle fibers (COX ×20). A large number of cells exhibited the reduced activities of complexes I, II, and IV.

The analysis of a panel of 368 nuclear genes involved in mitochondrial cytopathies highlighted two variants in the *ACAD9* gene: a c.1240C> T p.Arg414Cys class 5 variant, which is already described, and a c.1636G> A p.Val546Met class 3 or 4 variant, which has never been identified or reported (Figure 1A). This variant position has been reported as a change from valine to leucine. It is outside the ACAD family motif but belongs to the second Acyl-CoA dehydrogenase/oxidase C-terminal domain.

The family survey shows that the variant p.Arg414Cys is inherited from the mother and the variant p.Val546Met from the father. Our index case is therefore a compound heterozygous, reinforcing the diagnosis of myopathy sensitive to riboflavin and autosomal recessive transmission.

The muscle biopsy showed clear abnormalities in favor of mitochondrial cytopathy: ragged red fibers on GT and HE, large white negative COX fibers, and the accumulation of lipid droplets visualized by ORO coloration in few cells (Figure 1B). The respiratory chain study reveals a severe deficit of complex I with only 9% of residual activity. The study of mitochondrial DNA only revealed minimal deletions visible by “large-fragment” PCR. The search for pathogenic variants linked to the MERRF, MELAS, NARP syndromes and the high-throughput sequencing of mitochondrial DNA are non-contributory.

### 2.2. The Expression of V546M Variant Does Not Affect the Mitochondrial Efficiency

**Figure 2:**
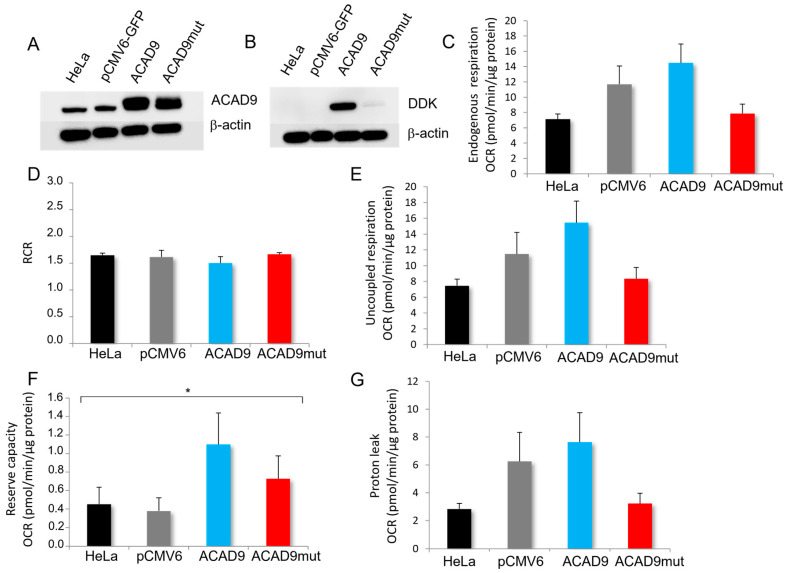
The effect of ACAD9 genetic variant V546M on mitochondrial respiration. (**A**) The Western Blot analysis shows the expression of ACAD9 protein in HeLa cells and HeLa cells transfected with the plasmid pCMV6-GFP, pCMV6-ACAD9-Myc-DDK or pCMV6-ACAD9mut. The protein content was normalized with the β-actin expression level. (**B**) The DDK tag expression was verified to distinguish endogenous ACAD9 from the protein overexpressed in ACAD9 and ACAD9mut cells. (**C**) The oxygen consumption rates (OCRs) were measured in the endogenous condition for non-transfected HeLa cells, the ones transfected with the empty plasmid pCMV6-GFP and the ones containing cDNA of ACAD9, wild-type pCMV6-ACAD9-Myc-DDK, or pCMV6-ACAD9mut for the variant V546M. The results are expressed in pmol/min/µg protein, and represent means ± standard error of the mean (SEM), with *N* = 4 experiments. (**D**) Respiratory control ratio (RCR) was determined by dividing the endogenous OCR by the respiration rate measured in the presence of 2.5 µg/mL oligomycin, a F_1_F_O_-ATP synthase inhibitor, for each condition. Each bar is a mean of ratios ± SEM. (**E**) The uncoupled respiration was assayed by adding 4 µM of CCCP. Each OCR bar value illustrates the mean ± SEM. (**F**) The reserve capacity was estimated by subtracting for each well the endogenous respiration rate from the uncoupled OCR. Each bar graph represents mean ± SEM, with * for *p* ≤ 0.05 (one-way ANOVA, Tukey’s multiple comparisons test). (**G**) The proton leak was calculated by deducting from each OCR value, obtained in presence of oligomycin, the remaining rates measured with antimycin. The results are expressed in mean ± SEM.

To investigate the impact of the new genetic variant V546M on mitochondrial functions, cells were first transfected with different plasmids. The overexpression of wild-type *ACAD9* was obtained with the plasmid pCMV6-ACAD9-Myc-DDK. Cells expressing the genetic variant V546M identified in the patient case were acquired with the plasmid pCMV6-ACAD9mut. The transfection efficiency was assayed by Western blot. The endogenous expression of ACAD9 was detected in all conditions, even in non-transfected HeLa cells and cells transfected with an empty plasmid pCMV6-GFP (Figure 2A).

As expected, the overexpression of ACAD9 was noticed in the cells transfected with pCMV6-ACAD9-Myc-DDK and ACAD9mut (Figure 2A,B). Then, the oxygen consumption rate (OCR) measurements were assessed to evaluate cellular respiration in these conditions. Even though the OCR measured in the endogenous condition seemed reduced in ACAD9mut cells when compared to ACAD9, the difference remains statistically non-significant (Figure 2C; *p* = 0.0529). The respiratory control ratio (RCR) that reflects the coupling between mitochondrial respiration and ATP production was estimated by normalizing the endogenous OCR with the rates obtained in the presence of oligomycin, so after the ATP synthesis inhibition (Figure 2D). The data show very similar ratios between the different conditions, suggesting a similar coupling degree or mitochondrial efficiency (1.50 ± 0.15 for ACAD9 and 1.67 ± 0.07 for ACAD9mut). The uncoupled respiration or maximal respiration was monitored in the presence of a protonophore, CCCP (Figure 2E). As observed for the endogenous respiration, the OCR values are slightly decreased in ACAD9mut cells when compared to ACAD9 cells, but again the variation remains statistically non-significant (*p* = 0.075). The reserve capacity assessed by calculating the difference between the uncoupled and endogenous respirations showed an increase in OCR in ACAD9 cells when compared to the two controls, i.e., non-transfected HeLa cells and cells transfected with the empty plasmid pCMV6 (Figure 2F, *p* = 0.0194). However, the rate values from ACAD9mut cells were between the OCR of the controls and that of ACAD9 cells. At last, the proton leak that reflects the permeability of the inner mitochondrial membrane to protons presented the same pattern as that of endogenous and uncoupled OCR ones (Figure 2G). It was determined by subtracting the OCR measured with antimycin from the OCR values in the presence of oligomycin. Together, the analysis revealed that the V546M variant alone is able to induce borderline changes regarding the mitochondrial respiration without affecting the coupling between the oxidation of the reducing equivalents by the respiratory chain and the phosphorylation of ADP for ATP synthesis.

To pursue the bioenergetics characterization of the cells and highlight an eventual impact of the new genetic variant, the ATP content was assayed. The cells transfected with the plasmid containing the wild-type *ACAD9* sequence and the ones overexpressing the mutated form both presented a similar ATP content (128.72% ± 24.48 and 105.97% ± 12.28, respectively). This result corroborates the absence of change in RCR, i.e., the mitochondrial efficiency.

### 2.3. The Expression of V546M Variant Reduces the Complex I Activity

**Figure 3:**
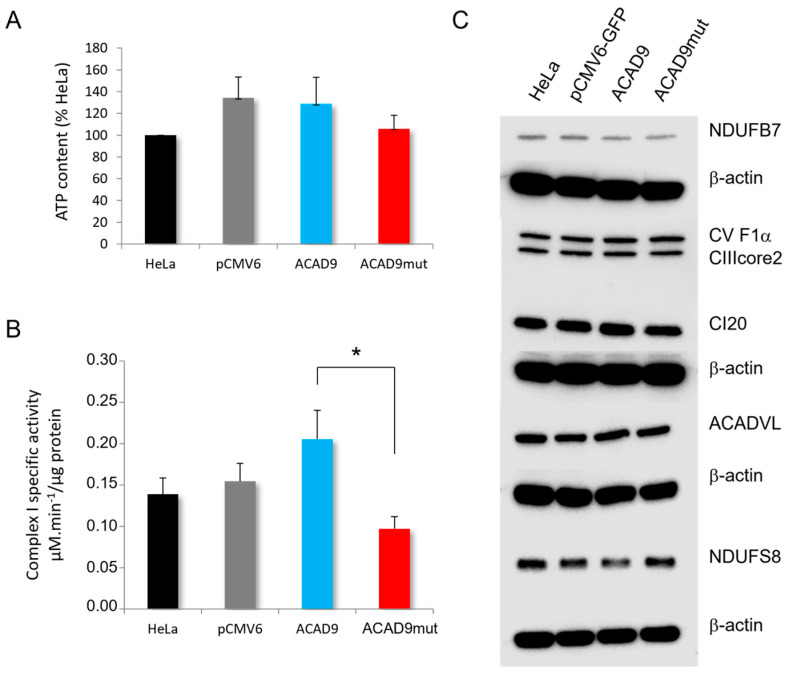
The impact of V546M variant on energy production and OXPHOS system activity. (**A**) The ATP content was quantified by luminescence for non-transfected HeLa cells (HeLa), cells transfected with the empty plasmid (pCMV6), and the ones overexpressing wild-type (ACAD9) or mutated ACAD9 (ACAD9mut). The results are expressed in percent of ATP value in non-transfected HeLa cells. Each bar graph illustrates mean ± SEM, with *N* = 3 experiments. (**B**) The complex I activity was assayed by spectrophotometry in µM.min^−1^/µg protein. Each specific activity value is mean ± SEM, with *N* = 4 experiments and * for *p* ≤ 0.05 (unpaired t test with Welch’s correction). (**C**) The protein expression level of subunits of complex I (NDUFB7, CI20, NDUFS8), ATP synthase (CV F1a), complex III (CIIIcore2), ACADVL, and β-actin were investigated by Western blot on 20 µg of proteins of each cell lysate.

Knowing that ACAD9 is involved in complex I assembly, the activity of this particular OXPHOS complex was measured spectrophotometrically. In Figure 3B, the data show a 52.91% decrease in the complex I specific activity in cells transfected with the mutant, when compared to cells overexpressing wild-type ACAD9 (0.097 µM.min^−1^/µg protein ± 0.0144 for ACAD9mut versus 0.206 ± 0.0347 for ACAD9, *p* = 0.0116).

Interestingly, the activity of the complex I in ACAD9mut cells was also reduced when compared to the cells transfected with the empty plasmid pCMV6 (0.154 ± 0.0219, *p* = 0.0471).

To verify whether the lower complex I activity observed in the case of the expression of the genetic variant V546M was due to a decrease in the protein content, the amount of complex I was analyzed by Western blot on the different cell lysates. The expression of NDUFB7, CI20, and NDUFS8, three subunits of the complex I, was not modified in ACAD9 and ACAD9mut cells compared to the controls (Figure 3C). Regarding the other OXPHOS complexes, no variation was observed for CV F1a and CIIIcore2 protein contents in the different cells. Furthermore, ACADVL, also known as ACAD6, remained the same following wild-type ACAD9 or V546M variant overexpression. Therefore, the inhibition of complex I activity triggered by this new genetic variant is revealed despite no change in complex I protein content and other OXPHOS complexes, suggesting a more likely remodeling of this complex.

**Figure 4:**
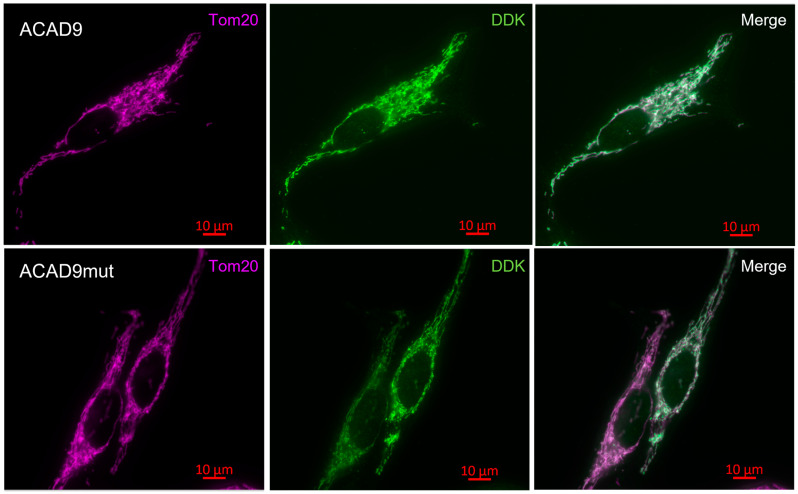
The effect of V546M variant on ACAD9 localization. HeLa cells transfected with the plasmid pCMV6-ACAD9-Myc-DDK or ACAD9mut were incubated with anti-tom20 (rabbit) and anti-DDK (mouse) primary antibodies following cell fixation with 4% PFA and permeabilization by 0.15% Triton X-100. The labeling of tom20 was visualized by fluorescence microscopy in Far-red with a goat anti-rabbit Alexa Fluor^®^647 and for DDK in green with a goat anti-mouse Alexa Fluor^®^488. The overlaps between the mitochondrial protein tom20 and wild-type or mutated ACAD9 appear in white.

At last, to pursue the characterization of V546M variant effects on mitochondria, the localization of ACAD9 and the mitochondrial network were investigated (Figure 4).

As expected, the mutation did not affect the mitochondrial localization of ACAD9 or any aspect of the mitochondrial network.

## 3. Discussion

The symptoms of this new patient reflected initially a mitochondrial cytopathology that was confirmed by the diagnosis made possible with the anatomopathology and molecular biology analysis. On a 368 genes’ panel, the genetic analysis permitted the identification of two mutations on the *ACAD9* gene [22,23,24]. As a compound heterozygote, the first mutation has already been described in the literature [3]. The mutation p.Arg414Cys responsible for progressive encephalomyopathy in a homozygous patient has been previously reported to induce a decrease in complex I and III activities. The protein content analysis by Western blot showed a massive reduction in complex I activity in the fibroblasts and muscles of the patient. Muscle weakness and exercise intolerance were the clinical criteria for this patient. In another patient case, the *ACAD9* homozygous mutation p.Arg414Cys with mitochondrial DNA deletions was responsible for a mild phenotype with severe impairment in complex I assembly [6]. On the other hand, the precise variant p.Val546Met was not previously revealed. However, homozygote patients with the variant p.Val546Leu presented cardiomyopathy with a reduction in complex I activity in muscle tissue or lymphocytes [21]. In addition, more than 100 genes have been reported to be involved in the various pathological cardiac remodeling. Cardiomyopathy is complex because different mutations in one gene are able to induce different phenotypes [25]. ACAD9 deficiency is usually associated with cardiac hypertrophy [12,21,26,27,28]. To focus on the genetic causes of hypertrophic cardiomyopathy, the reported genes are mainly involved in the structural organization of the cardiac tissue. Interestingly, genes coding for mitochondrial proteins, such as *TAZ*, *DNAJC19,* or *SDHA,* were responsible for dilated cardiomyopathy. On the other hand, ACAD9 is a mitochondrial protein involved in the β-oxidation of fatty acids and in complex I assembly. Thus, alterations in this metabolic pathway or in complex I function are usually triggered in ACAD9 deficiency [29,30,31,32]. The observed cardiomyopathy may be consecutive to the mitochondrial alterations.

In this particular case, the compound heterozygote patient presented a remaining complex I activity of 9%. The histochemical analysis also revealed ragged red fibers, muscle fibers negative for Cox activity, an increase in lipid droplets indicating alterations in mitochondrial functions, more precisely on the respiratory chain (at the complexes I and IV), and impairments in fatty acid oxidation because of the accumulation of neutral lipids observed with oil red [33]. Deletions of mitochondrial DNA were also reported; however, no variant was associated with MERRF, MELAS, or NARP. The patient was also treated with a 1 g vial of L-carnitine at a rate of 7 vials a day for 5 years. However, this treatment was ineffective according to the patient, and poorly tolerated after this length of time. For the last 3 years, it was combined with 50 mg of ubidecarenone (coenzyme Q10) 3 times a day. Eventually, all treatments were progressively discontinued. They were occasionally re-administered to relieve certain symptoms. The treatment of choice in this context was riboflavin (vitamin B2).

**Figure 5:**
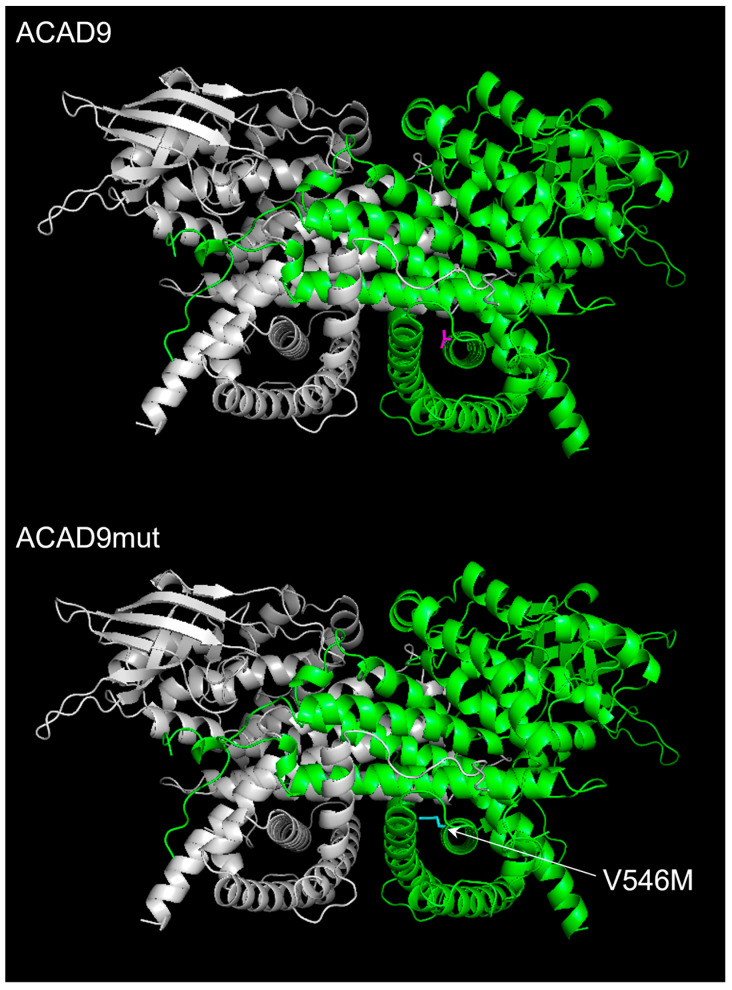
The structural organization of ACAD9. The wild-type protein ACAD9 is a homodimer represented with the software Pymol 3.1 from the PDB sequence 8PHE obtained by electron microscopy with a resolution of 3.1Å [29]. One monomer is colored in gray and the other one in green. For simplification, the visualization of the valine’s lateral chain at position 546 is shown in one monomer in magenta. For ACAD9mut, the mutation is illustrated in blue, with the lateral chain of methionine.

The approach used in our study was totally adequate to investigate the effect of this new *ACAD9* mutation. By overexpressing V546M in HeLa cells, the direct impact on mitochondrial functions could be revealed. Cybrids are a cell model that is usually used to investigate the impact of mtDNA mutations on mitochondrial functions. They are generated with cells deprived of mitochondria, r0 cells, and platelets from patients with mitochondrial cytopathology [34]. The fusion of these two cells permits us to obtain a cybrid with a known or controlled nuclear DNA and the mitochondrial pool of a patient. In other words, cybrid generation allows to abstract the nuclear genetic background from mtDNA mutations. In our case, the purpose was to highlight the relevance of the *ACAD9* mutation, a gene which is located in the nucleus. Therefore, there is no advantage to use the cybrid approach to decipher the pathogenicity of the V546M variant. The strategy to express the V546M variant in a cell model was necessary to clearly identify the effect of this mutation. The investigation permitted us to conclude that V546M does not affect the mitochondrial efficiency because of no change in the RCR. This result is in accordance with the ATP content, which was similar in cells overexpressing ACAD9 and the ones with ACAD9mut. The functionality of the respiratory chain remained unchanged regarding endogenous and uncoupled OCRs. The reserve capacity (i.e., the difference between the uncoupled respiration and the endogenous one) was higher in ACAD9-overexpressing cells when compared to the controls. However, no significant change was observed between ACAD9mut-expressing cells and the controls, even with ACAD9. This phenomenon can be explained by the fact that increasing the expression of ACAD9 might be able to promote an increase in the amount of complex I. This point was not illustrated with the Western blot results. The content of CI20 subunit remained similar in ACAD9-overexpressing cells when compared that of other cells. The same observation was made with ACAD9mut-expressing cells. The content of two distinct complex I subunits was also investigated, and neither NDUFB7 nor NDUFS8 were modulated in ACAD9 or ACAD9mut cells. Overall, the Western blot analysis indicated that selected subunits of the OXPHOS system remained the same in these two cell lines. However, the V546M variant was able to significantly reduce complex I activity compared to ACAD9-overexpressing cells and the control pCMV6. Thus, in a structural point of view, a longer lateral chain at the AA546 (Figure 5) in the C-terminal domain of the protein is enough to alter the function of ACAD9 and probably the complex I subunit composition instead of its total amount.

NDUFB7 and CI20 (also known as NDUFB8) are parts of the ND5 module complex I [30]. And NDUFS8 is part of the Q module. Thus, our results suggest that the V546M variant has no impact on the composition of these two modules within the complex I. Thereby, knowing that ACAD9 normally contributes to the assembly of the ND2 module with Q+ND1 modules, a remodeling triggered in the case of V546M may be located in these associated modules. Before trying to explain structural changes that affect mitochondrial complex I, an aspect must be kept in mind that ACAD9 is a homodimeric protein. Thus, when ACAD9mut is overexpressed in HeLa cells, there is a high probability that each monomer carries the V546M mutation. Thus, the dimeric mutant is the preponderant form. However, the wild-type dimer and the protein with only one mutated monomer should be expressed in a small amount. In the patient case with the compound heterozygosity, most of the ACAD9 protein may be constituted with one monomer with the variant R414C and the other with the V546M variant. Nevertheless, a small amount of ACAD9 content may carry twice the same mutation, such as R414C or V546M in each monomer. Thus, considering the pool of ACAD9-modified proteins in a patient muscle cell, or a cardiomyocyte, the activity of the complex I is reduced to a larger extent than that in the cell model generated here, which is consistent with the histoenzymology results (9% of residual activity). Moreover, the presence of mtDNA deletions revealed in the patient case may exacerbate the mitochondrial phenotype.

In conclusion, we have demonstrated here the pathogenicity of the V546M variant and its impact on the specific activity of complex I, which is halved.

## 4. Materials and Methods

### 4.1. Patient Description/Diagnosis Arguments

#### 4.1.1. Clinical Description

We identified the case of a patient from Martinique affected by a myopathy sensitive to riboflavin with the presence of 2 variants of the *ACAD9* gene, one of which has never been described but reported as a conflicting classification of pathogenicity. She consulted a clinic for fatigue on exertion, for the first time at the age of 36. Clinically, the examination found scoliosis and mild hypertrophic cardiomyopathy. She suffers from menopause and high blood pressure treated since the age of 35. Since childhood, this patient had presented a maladaptation to exertion, with abnormal rapid exhaustion for moderate exertion. Later, she developed myoclonic epilepsy with bilateral electroencephalogram polyspike and waves. Moreover, moderate left ventricular hypertrophy and hypoacusis were detected. No visual nor oculomotor disorders were noticed.

In her personal history, we noted transient insulin-dependent diabetes in childhood, an epilepsy attack at the age of 15, irregular menstruation, fertility disorders most certainly connected with endometriosis discovered at the age of 30, and the removal of an atrophied kidney.

**Figure A1:**
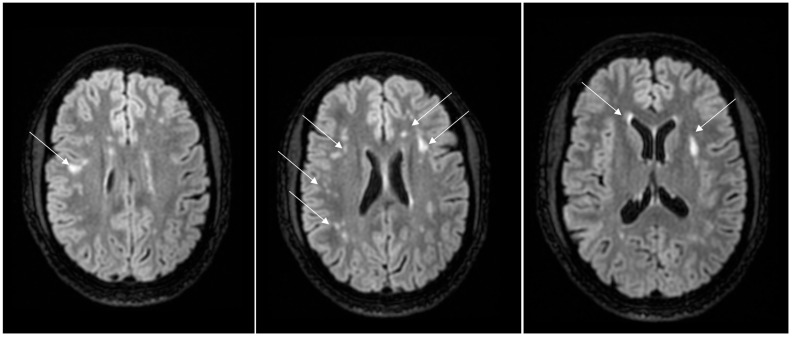
Brain MRI in ACAD9 deficiency. The compound heterozygote patient’s MRI shows FLAIR hypersignals (see arrows) in the subcortical and periventricular areas.

The signs worsened, with the appearance of swallowing disorders and false routes, chronic headache, exertional dyspnea, episodes of “letting go of objects” linked to distal muscle weakness, and memory problems. The Measurement of Motor Function was 98.96%, and the walking perimeter was 100 m and required repeated stops. CPK and the lactate/pyruvate ratio were moderately increased. Brain MRI showed non-specific subcortical and periventricular FLAIR hypersignals with unusual stroke-like appearance expected in mitochondrial cytopathies. Those are signs of demyelination (Figure A1).

#### 4.1.2. Histological Analysis of Muscle Biopsy

An open-muscle biopsy was performed on the left deltoid after obtaining informed consent. For conventional histochemical techniques, 10 mm-thick cryostat sections were stained with hematoxylin and eosin (HE), modified Gomori trichrome (GT), oil red O (ORO), reduced nicotinamide adenine dinucleotide dehydrogenase–tetrazolium reductase (NADH-TR), succinic dehydrogenase (SDH), and cytochrome oxidase (COX).

Digital photographs of each section were obtained with an AxioCam HRc camera linked to an Axioplan brightfield microscope (Zeiss, Jena, Germany) and processed with the AxioVision 4.4 software (Zeiss).

### 4.2. In Vitro Analysis

All the reagents used in this study were purchased from Sigma Aldrich (Saint-Quentin-Fallavier, France), with the exception of the ATP Assay Kit and Fugene^®^ HD (Promega, Charbonnières-les-Bains, France).

#### 4.2.1. Cell Culture

HeLa cells were purchased from *ATCC*. They were grown in Dulbecco’s Modified Eagle Media (DMEM), *GIBCO*, containing 5.56 mM glucose, supplemented with 10% fetal calf serum and 100 U/mL penicillin and streptomycin. The cells were cultured in flasks and kept in an incubator with 5% CO_2_ at 37 °C. For the different experiments, cells were collected at 80% confluency in the exponential growth phase.

#### 4.2.2. Site-Directed Mutagenesis

**Figure A2:**
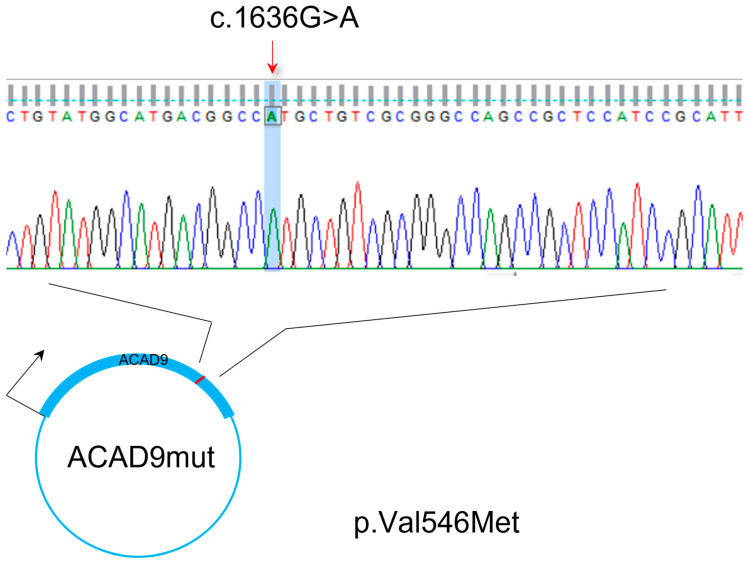
ACAD9mut plasmid representation. The cDNA of *ACAD9* in the plasmid pCMV6-Myc-DDK was modified by a single-nucleotide exchange during mutagenesis. A portion of the electropherogram shows the mutation site c.1636G>A responsible for the p.Val546Met *ACAD9* variant.

The *ACAD9* nucleotide variant c.1636G>A (p.Val546Met) detected in the patient was generated on a pCMV6-Myc-DDK plasmid containing *ACAD9* cDNA (OriGene, Rockville, MD, USA), with a QuikChange II XL Site-Directed Mutagenesis Kit (Agilent, Les Ulis, France), by following the manufacturer’s instructions. The sequences of the oligonucleotides’ primers used to insert the point mutation were ^5′^GGCATGACGGCCATGCTGTCGCGGG^3′^ for g1636a and ^5′^CCCGCGACAGCATGGCCGTCATGCC^3′^ for Rev. After the XL10-Gold competent bacterial transformation with the plasmid containing wild-type ACAD9 cDNA or the one with the single-mutation ACAD9mut, cells were spread on Petri dishes coated with LB agar containing kanamycin. A bacterial clone was collected from each Petri dish, and grown in LB medium supplemented with kanamycin overnight at 37 °C. Then, to collect the transformed bacteria, cell suspensions were centrifuged at 6000 rpm for 15 min at 4 °C. Each plasmid was isolated from a bacterial pellet by using a maxiprep NucleoBond Xtra Maxi endotoxin-free plasmid DNA kit (Macherey-Nagel, Hoerdt, France). The eluted plasmids were sequenced to confirm the mutagenesis (Genewiz, Leipzig, Germany), as schematized in Figure A2.

#### 4.2.3. Western Blot

Cell lysis was performed by using RIPA buffer for one hour on ice. After protein quantity determination with a Pierce BCA kit (Thermo Fisher Scientific, Courtaboeuf, France), the samples were diluted into a Laemmli buffer (Bio-Rad, Marnes-la-Coquette, France) with 4% β-mercaptoethanol. The samples were loaded in a 4–15% SDS polyacrylamide gradient mini gel (Bio-Rad) at 150 V. Proteins were transferred to a 0.2 µm polyvinylidine difluoride (PVDF) membrane, with a Turbo Trans-blot system. Membranes were blocked for one hour in 5% milk–PBS containing 0.05% Tween, and incubated overnight with primary antibodies. Antibodies against ACAD9 were obtained from ABclonal. Antibodies against DDK were purchased from Novusbio. The total OXPHOS complexes antibody cocktail was purchased from abcam and the antibody against β-actin from SIGMA. After three washes with PBS–0.05% Tween 20, the membranes were incubated for 1 h with horseradish peroxidase-conjugated goat anti-rabbit (Bio-Rad) or goat anti-mouse antibody diluted in 5% milk–PBS. At the end of the three last washes with PBS–Tween, the secondary antibody was detected with an Odyssey XF Imager (Li-COR, Bad Homburg, Germany) using the chemiluminescent SuperSignal™ West Pico PLUS Chemiluminescent Substrate (Pierce). The signal was quantified by densitometric analysis using the Image J 1.54p (NIH, Bethesda, MD, USA) software.

#### 4.2.4. Oxygen Consumption Rate Measurements

To monitor the mitochondrial oxygen consumption, cells were seeded in a seahorse 96-well plate. After an overnight incubation, cells were transfected for 48 h with the plasmids pCMV6-GFP, pCMV6-ACAD9-Myc-DDK, or ACAD9mut by using Fugene HD. The culture media was replaced by the assay medium composed of DMEM with 5.56 mM glucose, 1 mM pyruvate, 2 mM glutamine, and 1% penicillin–streptomycin without serum and bicarbonate. After 1 h of incubation at 37 C without CO_2_, the oxygen consumption rates (OCRs) were measured in the endogenous conditions with a XF96 extracellular flux analyzer (Seahorse Bioscience, Agilent, Les Ulis, France) at 37 °C. The respiratory control ratio (RCR) was determined by using oligomycin (2.5 μg/mL) for F_1_F_O_-ATP synthase inhibition. The maximal respiration also known as the uncoupled OCR was measured with 4 μM of CCCP. Thus, the reserve capacity also was estimated. The cells were then treated with 2µM of antimycin A, an inhibitor of complex III, in order to reveal the non-mitochondrial respiration. Following each experiment, the medium was removed from the wells, and replaced by RIPA in order to lyse the cells for 1 h at 4 °C. The protein content was determined in several wells for normalization by using the BCA kit. The OCR data are expressed in pmol/min/µg protein.

#### 4.2.5. ATP Assay

To assay their ATP content, the cells were seeded in a white 96-well plate with a clear bottom and 5000 cells per well. On the following day, cells were transfected with the plasmids pCMV6-GFP, pCMV6-ACAD9-Myc-DDK, or ACAD9mut by using Fugene HD. The intracellular ATP content was measured 48 h later by using the bioluminescent CellTiter-Glo 2.0 assay (Promega). A 50 µL volume of reagent was added to each well, and the cells were shaken for 2 min at room temperature (RT). Then, by the end of 10 min of incubation at RT, the bioluminescence was read in RLU (relative light unit). Standardization was performed with known quantities of standard ATP in the same conditions. The ATP content in each well was normalized by the protein quantity. Finally, data are expressed in percent of ATP amount in HeLa cells.

#### 4.2.6. Enzymology

Complex I specific activity was determined on different cell homogenates after 3 cycles of freezing/thaw. The protein content was estimated by the Pierce BCA kit. Then, the enzyme activity was measured spectrophotometrically at 340 nm and 37 °C for 70 µg of proteins, with 25 mM potassium phosphate, 3.75 mg/mL BSA, 100 µM decylubiquinone, and 100 µM NADH, in the presence or absence of rotenone (12.5 µM). The results are expressed in µM/min/µg of proteins.

#### 4.2.7. Immunocytochemistry

HeLa cells were transfected in a 4-well plate (Labtek, Thermo Fisher Scientific) with the plasmid pCMV6-ACAD9-Myc-DDK or ACAD9mut by using Fugene^®^ HD. After 48 h of transfection, the cells were fixed with 4% paraformaldehyde (PFA), then washed with PBS, and permeabilized with a 0.15% Triton X-100 solution. After 15 min of incubation, the triton solution was removed and replaced by a 10% BSA (bovine serum albumin) blocking solution. Following 45 min of blocking, HeLa cells were incubated for 2 h into a solution of primary antibody prepared in 10% BSA solution, anti-DDK (1:1000, mouse, NBP1-71705, NOVUS Biologicals, Noyal-Châtillon-sur-Seiche, France), and anti-tom20 (1:500, rabbit, ab186735, Abcam, Cambridge, UK). The non-specific binding was removed with several washes in PBS. Then, transfected HeLa cells were incubated for 45 min in secondary antibodies: 1:500 goat anti-mouse Alexa Fluor^®^488, with excitation/emission wavelength of 493 nm/518 nm, for DDK labeling and goat anti-rabbit Alexa Fluor^®^647, with excitation wavelength at 650 nm and emission wavelength at 671 nm, for tom20 labeling. Finally, after a couple of washes with PBS, the labeling of ACAD9 within the cells was observed by fluorescence microscopy (Zeiss, AxioVision; Carl Zeiss, Rueil-Malmaison, France), with a 63× oil objective with the appropriate filter. The co-localization of wild-type or mutated ACAD9 (via DDK tag) with Tom20, a mitochondrial protein, was observed with the Carl Zeiss 4.8 software.

#### 4.2.8. Statistical Analysis

All the presented data correspond to the mean value of *N* experiments ± SD or SEM when specified, with *N* ≥ 3. The comparison of obtained data sets was performed with the Student’s *t* test, one-way ANOVA, or another appropriate statistical analysis using the Excel software (Microsoft) and GraphPad Prism 10 software (GraphPad Software). Data sets were considered statistically different when *p* < 0.05.

## 5. Conclusions

To date, ACAD9 deficiency, known as a recessive neuromuscular disease, is often characterized by early symptoms and even severe neonatal clinical signs such as cardiomyopathy and neurological and liver dysfunctions. We report a patient with an attenuated phenotype linked to a composite heterozygous status for the *ACAD9* gene: R414C class V and V546M class III or IV (non-described variant). Homozygous patients reported with R414C variant are affected by cerebral development disorder and muscle dystrophy.

On the other side, homozygous patients with the V546L mutation presented cardiomyopathy as neonatal alterations that may be associated with left ventricular repolarization disorders. Overall, the compound heterozygous patient presented a mild and late-onset phenotype with fatigue on exertion, mild hypertrophic cardiomyopathy, high blood pressure, transient insulin-dependent diabetes, and epilepsy attacks.

Finally, V546M the variant of uncertain significance, recently identified in a patient, is demonstrated to be deleterious for the first time in this study. The compound heterozygous status seems to explain the attenuated phenotype in this particular case of ACAD9 deficiency, even though multiple deletions are present within mitochondrial DNA.

## Data Availability

Data is contained in the article.

## Abbreviations

The following abbreviations are used in this manuscript:

ACAD9	Acyl-CoA dehydrogenase 9
OMIM	Online Mendelian Inheritance in Man
ATP	Adenosine triphosphate
FAO	β-oxidation of fatty acids
SCAD	Short-chain fatty acid dehydrogenase
MCAD	Medium-chain fatty acid dehydrogenase
LCAD	Long-chain fatty acid dehydrogenase
VLCAD	Very-long-chain acyl-CoA dehydrogenase
FAD	Oxidized form of flavine adenine dinucleotide
NDUFAF1	NADH: Ubiquinone Oxidoreductase Complex Assembly Factor 1
ECSIT	Evolutionarily Conserved Signaling Intermediate in Toll Pathway
MCIA	Mitochondrial complex I intermediate assembly
TMEM126B	Transmembrane protein 126B
TMEM186	Transmembrane protein 186
TIMMDC1	Translocase of inner mitochondrial membrane domain containing 1
OXPHOS	Oxidative phosphorylation
NADH	Reduced form of nicotinamide adenine dinucleotide dehydrogenase
FADH_2_	Reduced form of flavin adenine dinucleotide
RIPA	Radioimmunoprecipitation assay buffer
BCA	Bicinchoninic acid
CPK	Creatine phosphokinase
HE	Hematoxylin and eosin
GT	Gomori trichrome
ORO	Oil red O
NADH-TR	Reduced nicotinamide adenine dinucleotide dehydrogenase–tetrazolium reductase
SDH	Succinic dehydrogenase
COX	Cytochrome oxidase
ATCC	American Type Culture Collection
OCR	Oxygen consumption rates
CCCP	Carbonyl cyanide m-chlorophenyl hydrazone
GFP	Green fluorescent protein
PBS	Phosphate-buffered saline
SEM	Standard error of the mean
MERRF	Myoclonic epilepsy with ragged red fibers
MELAS	Mitochondrial encephalomyopathy, lactic acidosis, and stroke-like episodes
NARP	Neuropathy, ataxia, and retinitis pigmentosa
NDUFB7	NADH: Ubiquinone Oxidoreductase Subunit B7
NDUFB8	NADH: Ubiquinone Oxidoreductase Subunit B8 (also known as CI20)
CI20	Complex I subunit of 20KDa
NDUFS8	NADH: Ubiquinone Oxidoreductase Subunit S8
ACADVL	Gene of VLCAD protein
TAZ	Tafazzin
DNAJC19	DNAJ heat shock protein family member C19
